# Dieckol Inhibits Autophagic Flux and Induces Apoptotic Cell Death in A375 Human Melanoma Cells via Lysosomal Dysfunction and Mitochondrial Membrane Impairment

**DOI:** 10.3390/ijms232214149

**Published:** 2022-11-16

**Authors:** Min-Hee Jo, Yong-Tae Kim, Sun Joo Park

**Affiliations:** 1 Department of Chemistry, Pukyong National University, Busan 48513, Republic of Korea; 2 Department of Food Science & Biotechnology, Kunsan National University, Gunsan 54150, Republic of Korea

**Keywords:** dieckol, autophagy, apoptosis, cell death, melanoma, antitumor activity

## Abstract

Dieckol is a natural brown algal-derived polyphenol and its cytotoxic potential against various types of cancer cells has been studied. However, the effects of dieckol on autophagy in cancer cells remain unknown. Here, we show that dieckol inhibits the growth of A375 human melanoma cells by inducing apoptotic cell death, which is associated with lysosomal dysfunction and the inhibition of autophagic flux. Dieckol induces autophagosome accumulation by inhibiting autophagosome-lysosome fusion. Moreover, dieckol not only triggers lysosomal membrane permeabilization, followed by an increase in lysosomal pH and the inactivation of cathepsin B and D, but also causes the loss of mitochondrial membrane potential. Importantly, a cathepsin D inhibitor partially relieved dieckol-induced mitochondrial membrane impairment and caspase-mediated apoptosis. Collectively, our findings indicate that dieckol is a novel autophagy inhibitor that induces apoptosis-mediated cell death via lysosomal dysfunction and mitochondrial membrane impairment in A375 human melanoma cells. This suggests the novel potential value of dieckol as a chemotherapeutic drug candidate for melanoma treatment.

## 1. Introduction

Melanoma is a malignant tumor that causes the most deaths among skin cancers that develop from melanocytes, which produce melanin pigment [1]. If not treated successfully at an early stage, melanoma rapidly grows and spreads to nearby lymph nodes or other organs in the body. Once metastasis progresses, the prognosis is very poor [2]. High genetic mutations in specific genes such as oncogenic *BRAF* have been discovered in patients with melanoma [3], hence, the importance of developing targeted therapies against BRAF signaling has increased. Inhibitors targeting BRAF and its downstream MEK, such as vemurafenib and trametinib, respectively, have been developed [4,5,6]. However, after a short period of remission, most melanomas acquire drug resistance and relapse [7,8]. Therefore, there is still a strong need for new effective therapeutic strategies or agents to combat malignant melanoma. 

Autophagy is a physiological cellular process that maintains cellular homeostasis by packaging unnecessary or dysfunctional cellular components into autophagosomes in which they are degraded in a lysosome-dependent manner [9,10]. Evidence suggests that dysregulation of autophagy is implicated in various human diseases, including cancer, lysosomal disorders, inflammation, and neurodegeneration [10,11]. In particular, the role of autophagy in cancer is very complex and seems to differ depending on the cancer type, progression state, genetic mutation, and metabolic sensitivity [10]. Autophagy is involved in both the survival and death of cancer cells [12,13]. Despite its inhibitory role in tumor initiation, emerging evidence has shown that autophagy contributes to the growth of malignant melanoma cells [14,15,16]. Therefore, the inhibition of autophagy may be a therapeutic strategy for the treatment of metastatic melanoma. 

Many efforts have been made to find anticancer components from marine algae, because marine environments provide biodiversity and an excellent source of bioactive compounds [17,18]. Dieckol (DK), a polyphenol isolated from some brown alga species, such as *Ecklonia cava*, has been reported to have various pharmacological properties, including anticancer, antioxidant, anti-inflammatory, and antiviral effects [19,20,21,22]. Dieckol is known to inhibit tumor cell growth by inducing apoptosis in cancer cells, such as breast cancer, lung cancer, colon cancer, pancreatic cancer, osteosarcoma, and leukemia cells [23,24,25,26,27,28]. 

However, the effects of dieckol on autophagy and autophagy-associated tumor cell growth remain to be determined. In this study, we examined the antitumor effect of dieckol in A375 human melanoma cells and determined whether its antitumor effect was associated with autophagy regulation. Our results indicate that dieckol not only inhibits autophagic flux by inhibiting autophagosome-lysosome fusion but also induces lysosomal dysfunction and mitochondria membrane impairment, which results in growth inhibition of A375 melanoma cells. 

## 2. Results

### 2.1. Dieckol Decreases Cell Growth and Colony Formation of A375 Human Melanoma Cells

**Figure 1:**
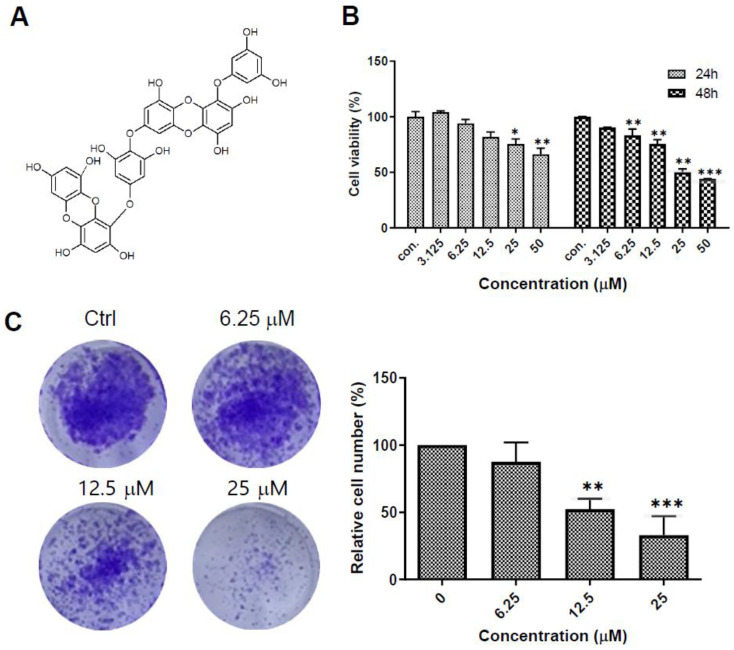
Dieckol (DK) suppresses proliferation and colony formation of A375 human melanoma cells. (**A**) Chemical structure of the DK molecules. (**B**) A375 cells were treated with indicated concentration of DK for 24 h or 48 h and the cell viability was measured by MTT assay. Results are shown as mean ± standard deviation (SD) of triplicate independent experiments. (**C**) Representative images of the colony formation assay and a bar graph showing the colony area. Results are shown as mean ± SD. *** *p* < 0.001, ** *p* < 0.01, * *p* < 0.05 compared to the control.

To examine the effects of dieckol on the growth of human melanoma cells, we treated A375 cells with various concentrations of dieckol and determined the viability of the cells and the ability of a single cell to grow into a large colony using the 3-(4,5-dimethylthiazol-2-yl)-2,5-diphenyltetrazolium bromide (MTT) assay and an in vitro colony formation assay. Dieckol decreased cell growth, with an IC_50_ value of 28.74 μM at 48 h (Figure 1B), and the colony-forming ability of the cells in a dose-dependent manner (Figure 1C). 

### 2.2. Dieckol Induces Caspase-Mediated Apoptosis in A375 Cells

**Figure 2:**
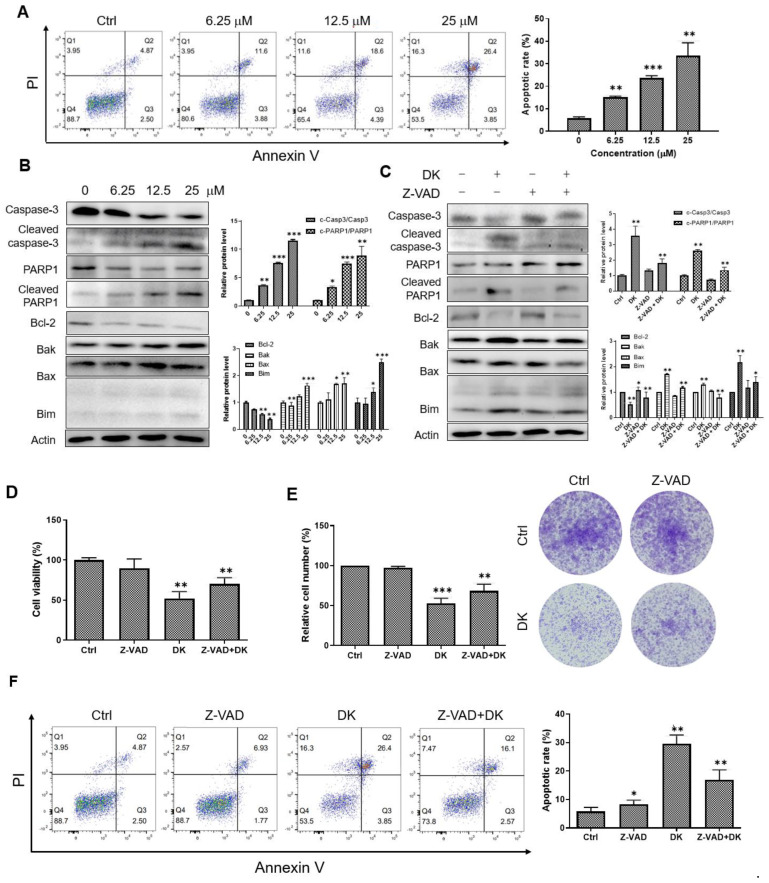
Dieckol (DK) induces apoptosis in A375 human melanoma cells. (**A**) Representative fluorescence-activated cell sorting (FACS) images showing DK-induced apoptosis. A375 cells were treated with vehicle DMSO or various concentration of DK for 48 h. The cells were double-stained with Annexin V-FITC and propidium iodide (PI). Apoptotic cell numbers were analyzed by flow cytometry. Annexin V-positive cells were described as apoptotic cells. The quantified results are shown as mean ± standard deviation (SD) of triplicate independent experiments. (**B**) Representative Western blot images showing the DK-induced apoptosis. A375 cells were treated with indicated concentration of DK for 48 h. The expression levels of caspase-3, cleaved caspase-3, poly-(ADP-ribose)-polymerase 1 (PARP1), cleaved PARP1, B-cell lymphoma-2 (Bcl-2), Bcl-2-associated X protein (Bax), Bcl-2 homologous antagonist/killer (Bak), Bcl-2 interacting mediator of cell death (Bim), and actin were analyzed by immunoblotting. Results are quantified and shown as mean ± SD of triplicate independent experiments. (**C**) A375 cells were treated with 25 μM DK in the presence or absence of Z-VAD-fmk (25 μM) for 48 h. The expression levels of indicated proteins were determined by immunoblotting and quantified by image J densitometric analysis. (**D**–**F**) Cells were treated the same as in (**C**). Cell viability, colony formation ability, and Annexin-positive apoptotic cells were analyzed. Results are quantified and shown as mean ± SD of triplicate independent experiments. *** *p* < 0.001, ** *p* < 0.01, * *p* < 0.05 compared to the control.

We examined whether dieckol-induced A375 melanoma cell death is associated with apoptosis. The A375 cells were double stained with annexin V/propidium iodide (PI) and analyzed using flow cytometry to assess the apoptosis rate. Dieckol caused annexin-positive phosphatidylserine plasma membrane externalization and loss of membrane integrity underlying PI positivity. The percentage of annexin V/PI double-positive late apoptotic cells increased in a dieckol dose-dependent manner (Figure 2A). The effect of dieckol on apoptosis was further determined by assessing the levels of key proteins involved in apoptosis signaling. Dieckol treatment activated caspase-3 and poly (ADP-ribose)-polymerase 1 (PARP1). There was a significant increase in the cleaved caspase-3 and PARP1 in cells treated with dieckol (Figure 2B). In addition, dieckol decreased the level of the anti-apoptotic protein B-cell lymphoma-2 (Bcl-2) but increased the levels of proapoptotic proteins Bcl-2 homologous antagonist/killer (Bak), Bcl-2-associated X protein (Bax), and Bcl-2 interacting mediator of cell death (Bim). These results suggested that dieckol induces caspase-dependent apoptosis downstream of the Bcl-2 family. Thus, we treated the cells with dieckol in the presence or absence of the fan-caspase inhibitor Z-VAD-fmk (Z-VAD) (Figure 2C). As shown in Figure 2C, the addition of Z-VAD reduced the cleavages of caspase-3 and PARP1, decreased Bcl-2 protein levels, and increased Bak, Bax, and Bim protein levels induced by dieckol. Moreover, Z-VAD prevented partially dieckol-induced cell death and decrease in colony formation (Figure 2D,E). The flow cytometry results showed that dieckol-induced cell death was rescued by Z-VAD treatment (Figure 2F). These findings indicated that dieckol induces caspase-mediated apoptosis, which decreases the growth of A375 melanoma cells. 

### 2.3. Dieckol Blocks Autophagic Flux by Inhibiting Autophagosome-Lysosome Fusion

**Figure 3:**
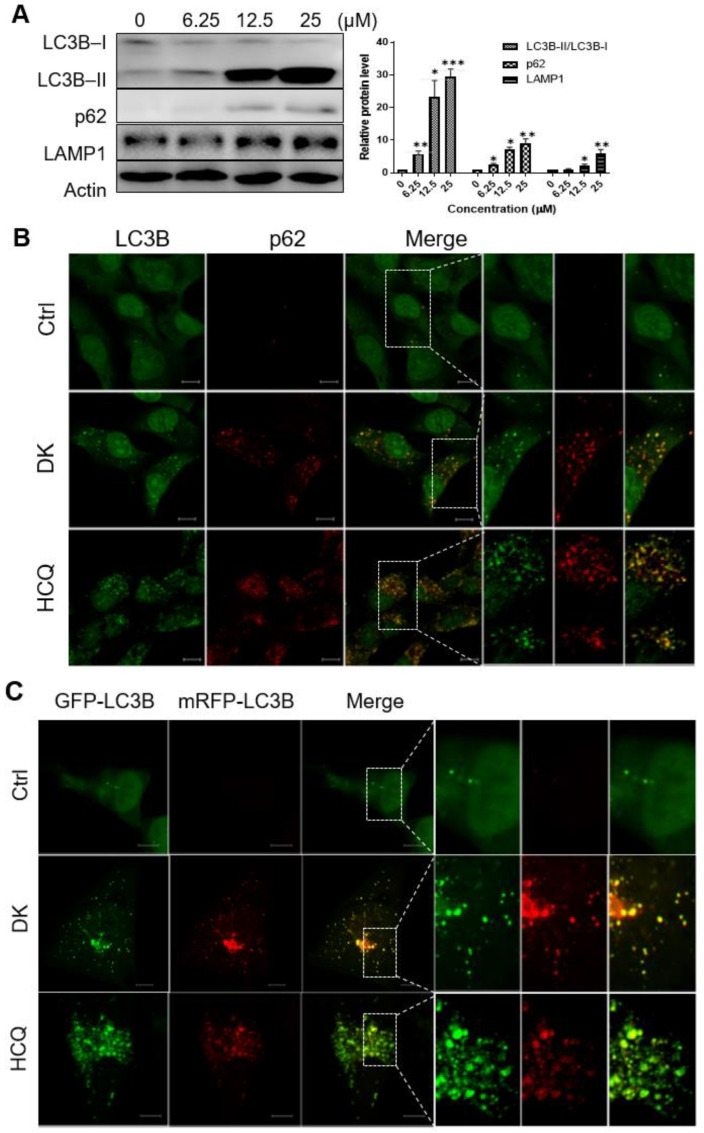

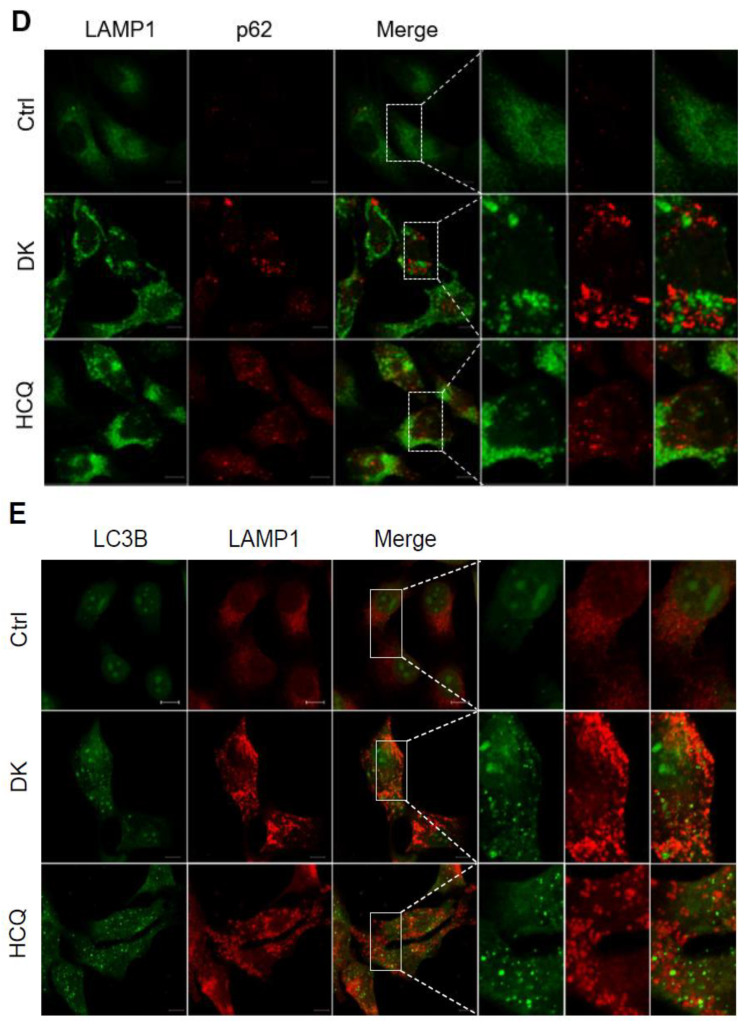
Dieckol (DK) blocks autophagic flux by inhibiting autophagosome-lysosome fusion. (**A**) A375 cells were treated with DMSO or various concentrations of DK for 48 h. The expression levels of microtubule-associated protein 1A/1B-light chain 3 (LC3B), sequestosome 1 (p62), and lysosome-associated membrane protein 1 (LAMP1) were detected by immunoblotting. (**B**) Representative immunofluorescence images showing the endogenous LC3B and p62. Cells were treated with DMSO or 25 μM DK for 48 h and then endogenous LC3B and p62 were visualized by immunofluorescence using primary antibodies, scale bar = 10 μM. Right panels depict enlarged images of the boxed areas seen in the left panels. (**C**) Representative immunofluorescence images showing colocalization of the GFP-LC3B and mRFP-LC3B by DK treatment. A375 cells were transfected with GFP-mRFP-LC3B. After 24 h, the cells were incubated with DMSO, 25 μM DK, or 15 μM HCQ for 48 h and then visualized with confocal microscopy, scale bar = 10 μM. (**D**,**E**) Cells were treated the same as in (**B**) and then endogenous LC3B or p62 and LAMP1 were visualized by immunofluorescence using primary antibodies. Right panels depict enlarged images of the boxed areas seen in the left panels. The scale bar = 10 μM. *** *p* < 0.001, ** *p* < 0.01, * *p* < 0.05 compared to the control.

Recent studies have suggested that autophagy is associated with cell death [29,30]. To investigate whether dieckol regulated autophagy in A375 melanoma cells, we examined the protein levels of two autophagy markers, microtubule-associated protein 1A/1B-light chain 3 beta (LC3B) and sequestosome 1 (p62) (Figure 3A). Immunoblotting results showed that dieckol treatment increased LC3B levels. Notably, the LC3B-II protein levels significantly increased in a dose-dependent manner with dieckol. During autophagy, cytosolic LC3-I is first converted to the phosphatidylethanolamine-conjugated LC3-II form and recruited to the autophagosomal membrane. In the late stage of autophagy, autophagosomes fuse with lysosomes to form autolysosomes, which are subsequently degraded by lysosomal proteases [9,10]. Therefore, an increase in the amount of LC3-II could be attributed to either induction of autophagy or inhibition of autophagic flux [31,32,33]. We measured p62 protein levels to elucidate the role of dieckol in autophagic flux. The p62 links LC3 and cargo substrates and is degraded in autolysosomes [9,34]. Therefore, p62 degradation is commonly used as an autophagy flux marker [35]. As shown in Figure 3A, dieckol treatment caused an increase in the amount of p62 indicating that dieckol inhibited autophagic flux but not autophagy induction. Immunofluorescence results of Figure 3B showed that endogenous LC3B was diffusely distributed in the cytoplasm and nucleus of control cells, whereas dieckol treatment significantly increased LC3B-positive puncta, a general marker for autophagosomes. Furthermore, the LC3B-positive puncta were colocalized with endogenous p62 that increased after dieckol treatment. These results indicated that dieckol treatment resulted in autophagosome accumulation by blocking autophagic flux in A375 cells. Similar results were observed in cells treated with hydroxychloroquine (HCQ), a late-stage inhibitor of autophagy (Figure 3B).

To confirm the effect of dieckol on autophagic flux, A375 cells were transfected with a plasmid encoding the membrane-localized protein monomeric red fluorescent protein (mRFP)-green fluorescent protein (GFP)-LC3B. The GFP fluorescent signal is easily quenched in the low pH lysosomes, whereas mRFP is stable even in acidic lysosomes. Therefore, if autolysosomal degradation does not occur normally, yellow puncta overlaying the red and green puncta are prominently observed. As shown in Figure 3C, we found increases in yellow punctuate fluorescence in dieckol-treated A375 cells compared to control cells, similar to that seen in HCQ-treated cells. These results indicate that dieckol treatment blocked autophagic degradation. 

Lysosome-associated membrane protein 1 (LAMP1), a major lysosomal membrane protein that plays a key role in maintaining lysosomal function and assisting the fusion between autophagosomes and lysosomes [36,37]. We found that the protein level of LAMP1 was increased by dieckol treatment (Figure 3A), whereas the increased LAMP1 was not colocalized with the LC3B- or p62-containing autophagosome puncta, similar to that in HCQ-treated cells (Figure 3D,E), indicating that dieckol impairs the fusion between autophagosomes and lysosomes, resulting in the inhibition of autophagic degradation and autophagic influx. These results support that an increase of LAMP1 in dieckol- or HCQ-treated cells is caused by the impaired autophagic lysosomal protein turnover. The HCQ, used as a positive control in this assay, has been known to decrease autophagosome-lysosome fusion and inhibit autophagic flux [38,39].

### 2.4. Inhibition of Autophagy Flux Confer Dieckol-Induced Cell Death in A375 Cells

**Figure 4:**
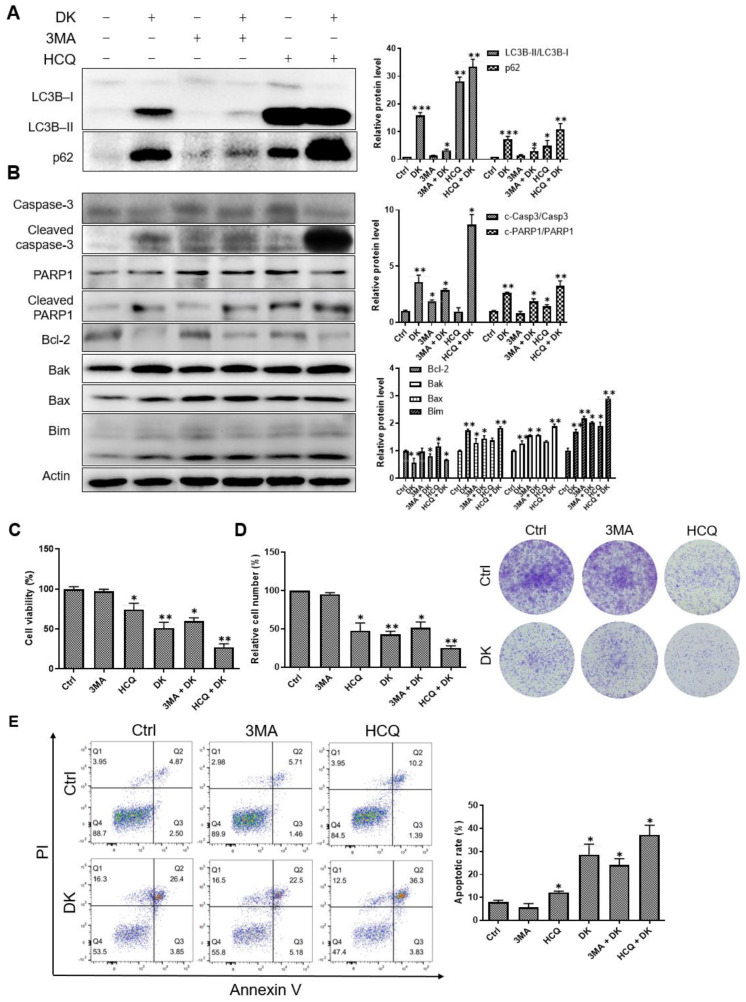
Inhibition of autophagy flux contributes to dieckol (DK)-induced cell death in A375 human melanoma cells. (**A**,**B**) A375 cells were treated with 3-methyladenine (3MA, 0.1 mM) or hydroxychloroquine (HCQ, 15 μM) in the presence or absence of 25 μM DK for 48 h, and cell lysates were analyzed by Western blotting for endogenous proteins. Representative images are shown. The expression levels of proteins were quantified by image J densitometric analysis. Results are shown as mean ± standard deviation (SD) of triplicate independent experiments. (**C**–**E**) Cells were treated the same as in (**A**). Cell viability, colony formation ability, and Annexin-positive apoptotic cells were analyzed. Results are quantified and shown as mean ± SD of triplicate independent experiments. *** *p* < 0.001, ** *p* < 0.01, * *p* < 0.05 compared to the control.

To examine whether inhibition of autophagic flux contributed to dieckol-induced cell death, A375 cells were treated with dieckol in the presence or absence of 3-methyladenine (3MA) or HCQ, an early- or late-stage inhibitor of autophagy, respectively. As shown in Figure 4A, significant increase in the protein levels of LC3B-II and p62 was detected in cells treated with dieckol or HCQ, but not in cells treated with 3MA. In a combination assay, although 3MA apparently reduced the dieckol-induced protein increase in LC3B-II and p62 (Figure 4A), 3MA had only a little effect on dieckol-induced not only activation of caspase-3 and PARP1 (Figure 4B) but also cell death and colony formation inhibition (Figure 4B–E). On the other hand, HCQ further enhanced the dieckol-induced accumulation of LC3B-II and p62, as well as the effect of dieckol on apoptosis regulatory proteins, caspase-3, PARP1, Bak, Bax, Bim, and Bcl-2 (Figure 4A,B). Furthermore, HCQ exhibited a synergistic effect with dieckol in inducing apoptotic cell death and inhibiting colony formation (Figure 4C–E). These data indicate that autophagy flux inhibition is associated with dieckol-induced cell death, although autophagosome accumulation alone is not sufficient for the cell death. It appears that dieckol-mediated inhibition at later stages of autophagy contributes mainly to dieckol-induced cell death. It is likely that dieckol acts primarily in post-autophagosome formation via a mechanism similar to that of HCQ.

### 2.5. Dieckol Induces Lysosomal Membrane Permeabilization (LMP) and Inhibits Lysosomal Protease Cathepsin Activity

**Figure 5:**
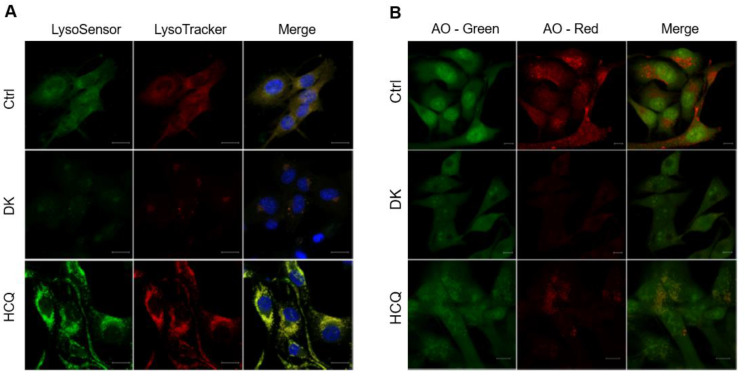
Dieckol (DK) induces lysosomal membrane permeabilization (LMP). (**A**) A375 human melanoma cells were treated with 25 μM DK or 25 μM HCQ for 48 h, followed by staining with LysoTracker red (100 nM) for 30 min and, with a LysoSensor green dye (2 μM) and Hochest 33,342 (15 μg/mL) for 10 min. scale bar = 10 μM. (**B**) Cells were treated the same as in (**A**) and LMP was measured by Acridine Orange (AO) staining under a fluorescence microscopy. Representative immunofluorescence images were shown, scale bar = 10 μM.

It is known that the blocking of autophagic flux is often associated with lysosomal dysfunction [40,41]. Therefore, we investigated the effect of dieckol on lysosomal function. We first examined whether dieckol affects lysosomal pH using the pH-sensitive dyes LysoSensor green and LysoTracker red, which stain acidic organelles, such as lysosomes. The addition of dieckol decreased the fluorescence intensity of dyes, indicating that dieckol caused a decrease in lysosomal acidity in the cells (Figure 5A). Next, we performed acridine orange (AO) staining to examine lysosomal membrane integrity. The AO, a lysosomotropic metachromatic fluorochrome, fluoresces red inside lysosomes and is weakly green in the cytosol. Figure 5B shows that dieckol treatment led to a significant loss of red fluorescence of AO in cells, consistent with the results of Lysotracker red staining, indicating that dieckol caused LMP in A375 cells. 

**Figure 6:**
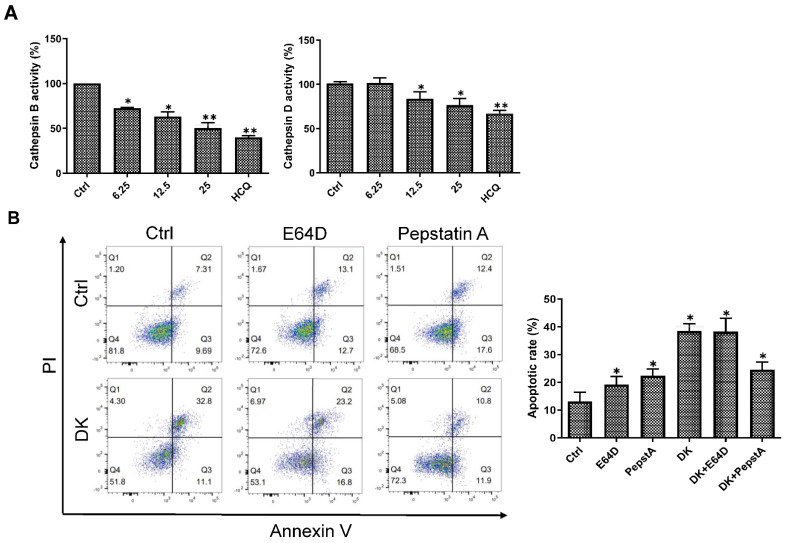
Dieckol (DK) inhibits cathepsins activity and pepstatin A, an inhibitor of cathepsin D (CTSD), restored DK-induced apoptosis. (**A**) A375 human melanoma cells were treated with various concentrations of DK or 15 μM HCQ for 48 h and the cell lysates were collected. Enzyme activity of CTSB and CTSD was analyzed using fluorogenic kits. Results are quantified and shown as mean ± standard deviation (SD) of triplicate independent experiments. (**B**) Cells were treated with E64D (50 μM) or pepstatin A (50 μM) in the presence or absence of 25 μM DK for 48 h. Apoptotic cells were analyzed by flow cytometry. Annexin V-positive cells were described as apoptotic cells. The quantified results are shown as mean ± standard deviation (SD) of triplicate independent experiments. ** *p* < 0.01, * *p* < 0.05 compared to the control.

Lysosomal membrane permeabilization is normally accompanied by a reduction in lysosomal acidification and release into the cytosol and inactivation of lysosomal proteases such as cathepsins [40,42]. Cathepsin B (CTSB) and cathepsin D (CTSD) are key mediators of lysosomal-mediated cell death and cathepsin inhibitors reduce human melanoma growth and lung metastasis [43,44,45,46]. Figure 6A shows that the activation of CTSB and CTSD obviously decreased in cells treated with dieckol, similar to that of HCQ. These findings suggest that dieckol-induced LMP and lysosomal deacidification results in the inactivation of acidic lysosomal protease cathepsins. To investigate whether dieckol-induced impairment of lysosomal membrane and leaked cathepsins are involved in dieckol-induced cell death, we treated A375 cells with the cathepsin inhibitor E64D or pepstatin A for CTSB or CTSD, respectively, in the presence of dieckol. As shown in Figure 6B, pepstatin A, an inhibitor of CTSD, protected dieckol-induced apoptotic cell death in A375 cells. This indicates that LMP and CTSD are involved in dieckol-induced apoptotic cell death. In this study, CSTB inhibitor E64D had little effect in the dieckol-treated cell death. Sufficient inhibition of CSTB by dieckol in cells might have diminished the effect of additional treatment with E64D. 

### 2.6. Dieckol Decreases Mitochondrial Membrane Potential (MMP)

**Figure 7:**
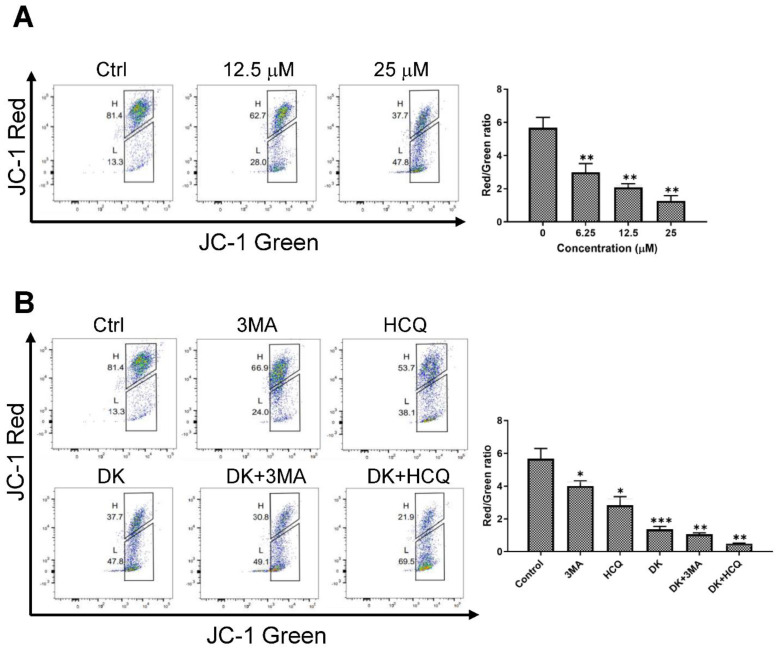

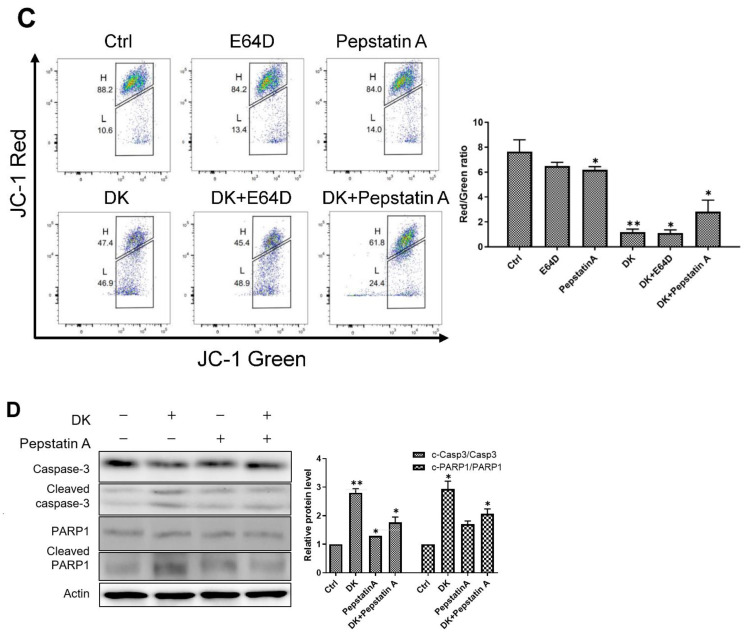
Dieckol (DK) decreases mitochondria membrane potential (MMP) and pepstatin A restored DK-induced MMP loss in cells. (**A**) A375 human melanoma cells were treated with various concentrations of DK for 48 h and the level of MMP were measured with JC-1 staining by flow cytometer analysis. Increased intensity of JC-1 green indicates decreased MMP. The intensity ratio of the green to red fluorescence was determined. Data is presented as the Mean ± standard deviation (SD). Representative images are shown. (**B**) Cells were treated with 3-methyladenine (3MA, 0.1 mM) or HCQ (15 μM) in the presence or absence of 25 μM DK for 48 h. The levels of MMP were measured with JC-1 staining as (**A**). (**C**) Cells were treated with E64D (50 μM) or pepstatin A (50 μM) in the presence or absence of 25 μM DK for 48 h. MMP level was measured with JC-1 staining. (**D**) The expression levels of caspase-3, cleaved caspase-3, PARP1, and cleaved PARP1 were also analyzed by immunoblotting. Results are quantified and shown as mean ± standard deviation (SD) of triplicate independent experiments. *** *p* < 0.001, ** *p* < 0.01, * *p* < 0.05 compared to the control.

Since LMP and lysosomal-leaked cathepsins are known to be involved in mitochondrial membrane dysfunction and caspase-dependent apoptotic cell death [47,48], we investigated whether dieckol affects MMP in cells. A375 cells were treated with dieckol and labeled with JC-1, a cationic fluorescent dye, to determine the MMP. In normal cells, JC-1 accumulates as an aggregate in the mitochondrial membrane, resulting in red fluorescence. The JC-1 is present in the green fluorescent monomeric form in damaged cells with diminished MMP. Dieckol decreased MMP in a dose-dependent manner (Figure 7A). In the combination assay, HCQ further enhanced dieckol-induced loss of MMP. 3MA had little effect on dieckol-induced loss of MMP (Figure 7B).

In addition, we found that pepstatin A, an inhibitor of CTSD, partially protected against dieckol-induced loss of MMP (Figure 7C) and attenuated activation of caspase-3 and PARP1 (Figure 7D). These findings suggest that dieckol causes rupture of the lysosome membrane and release of lysosomal CTSD into the cytosol. This induces mitochondrial membrane permeabilization and caspase-dependent apoptotic cell death signaling in A375 cells.

## 3. Discussion

Dieckol is a brown algal polyphenol compound with anticancer potential. The polyphenol inhibits the proliferation of various types of tumor cells including breast cancer [49], hepatoma cancer [24], ovarian cancer [50], osteosarcoma [51], and lung cancer [52]. Dieckol’s major mechanism of action against cancer cells has been interpreted invariably as the induction of apoptosis in cells [23,24,50], even though it was unsatisfactory. However, little is known about the effects of dieckol on autophagy in cancer cells. Recent studies have shown that the anticancer activity of several natural polyphenolic compounds, including quercetin, curcumin, and resveratrol, is associated with autophagy [53]. In most cases, polyphenols induce autophagy, causing cancer cells to survive or die [54,55,56,57,58]. For instance, in cancer cells that exhibit autophagic responses for survival, autophagy inhibitors prevent cancer progression and synergize with other pharmaceutical agents [56].

In the present study, we showed that dieckol treatment significantly increased the number of LC3B-positive autophagosomes in A375 human melanoma cells (Figure 3B). Dieckol inhibited autophagosome-lysosome fusion and autophagy flux (Figure 3C,D). An early-stage autophagy inhibitor, 3MA, markedly reduced dieckol-induced accumulation of LC3B-II and p62 (Figure 4A), but exhibited only a little effect on dieckol-induced MMP loss (Figure 7B) and apoptotic cell death (Figure 4B–E). In contrast, a late-stage autophagy inhibitor, HCQ, had effects similar to those of dieckol, and in a combination assay, enhanced the dieckol-induced autophagy flux inhibition (Figure 4A), MMP loss (Figure 7B), and cell death (Figure 4B–E). This indicates that dieckol might act at the late stage of autophagy, similar to HCQ. Interestingly, given that 3MA treatment alone did not affect the viability of A375 cells (Figure 4C–E), it seems that A375 melanoma cells maintain a low level of basal autophagy, and autophagy inhibition alone would not successfully achieve death of A375 cells. This may be supported by other data that reveals that BRAF inhibitor treatment of A375 cells stimulates induction of autophagy to avoid apoptosis [59,60].

Dieckol induces not only LMP but also inactivation of CTSB and CTSD. In addition, dieckol impairs MMP and induces caspase-mediated apoptosis of A375 cells. An inhibitor of CTSD decreased dieckol-induced MMP loss and caspase-mediated apoptotic cell death. Taken together, these data suggest that dieckol induces the first LMP and then the release of lysosomal acidic cathepsin D into the cytosol, which in turn induces MMP loss, followed by caspase activation and apoptosis.

Lysosomal cell death is commonly defined as a cell death pathway involving LMP and the release of lysosomal acidic cathepsins into the cytosol [47,61]. On the one hand, many studies have reported that the released cathepsins induce the activation of proapoptotic Bcl-2 family proteins, such as Bid and Bim, and the subsequent translocation of Bak and Bax into the outer mitochondrial membrane, which promotes loss of MMP and caspase-mediated apoptotic cell death [61,62,63]. On the other hand, some studies have shown that translocation of Bak and Bax into the lysosomal membrane directly induces LMP- and cathepsins-mediated autophagic cell death independently of MMP loss and caspase activation [64,65]. In our study, we found that dieckol increased Bim, Bak, and Bax and inhibitors of CTSD reversed the dieckol-induced MMP loss, caspase activation, and cell death. This suggests that dieckol-induced LMP and cathepsin D release might be followed by MMP loss-mediated apoptosis in A375 cells. However, we still cannot rule out the possibility of dieckol induced LMP/cathepsin-mediated autophagic cell death, independent of apoptosis, because the inhibitory effects of Z-VAD or pepstatin A on dieckol-induced cell death were both incomplete and partial. Therefore, further details of the mechanism by which dieckol induces LMP and the major factor linking LMP to MMP-mediated apoptosis remain to be elucidated to understand the mechanism of dieckol-induced cell death in A375 cells.

In the current study, we present that dieckol and HCQ have similar effects in some experiments related to autophagy in A375 cells. It is well known that HCQ, a derivative of chloroquine (CQ), is a lysosomal inhibitor with cytotoxic properties that block autophagy by inhibiting autophagysome-lysosome fusion [38]. We found that HCQ inhibited autophagic flux by inhibiting autophagosome-lysosome fusion in A375 cells. It also inhibited the activation of CSTB/CSTD, increased MMP loss and, decreased cell viability and colony formation in A375 cells, similar to dieckol. However, we also observed some differences in the cells exposed to dieckol or HCQ. The first is the difference in lysosomal acidity in both cells. We did not find a decrease in fluorescence intensity of LysoTracker red and LysoSensor green in HCQ-treated cells compared to that of the control, and even when a high concentration of more than 50 µM was used, the intensity was higher than that of the control cells, which might be explained by recent studies showing that the effect of CQ on lysosomal pH differs depending on the cell type and may be transient and instantly re-activated [38,66,67,68]. In addition, dieckol induced a dramatic decrease in AO red signal, indicating LMP, with short-term treatment, whereas long-term treatment with high concentrations of HCQ caused a progressive decline in the red staining of lysosomes with AO. Therefore, the mechanism of HCQ-induced cell death appears to be slightly different from that of dieckol. It is likely that HCQ functions primarily in blocking autophagy flux by impairing autophagosome–lysosome fusion rather than lysosomal dysfunction and mitochondria membrane impairment in A375 cells. Additional factors may be required for HCQ to trigger cell death in A375 melanoma cells. However, in our study, simultaneous treatment with dieckol and HCQ of A375 cells caused more pronounced cell death. Given that CQ and HCQ are the only drugs approved by the FDA for autophagy inhibition, dieckol may be a novel late-stage autophagy inhibitor and a key compound for human melanoma cancer therapy. The combined use of dieckol and HCQ may be a therapeutic method to cause more pronounced cell death in A375 melanoma cells.

Recent studies have shown that various cancer cells display high levels of autophagy to evade cell death, such as apoptosis, resulting in chemoresistance [69,70]. In the present study, we used A375 human melanoma cells harboring oncogenic mutant *BRAF^V600E^* [71]. Inhibition of *BRAF^V600E^* signaling for anticancer treatment has been shown to increase autophagy, promote tumor survival, and protect cancer cells from the cytotoxic effects of chemotherapy. Cells with BRAF inhibitor-resistance showed further increased autophagy compared to that in the responsive lines [72,73,74]. Therefore, dieckol may be effective as an anti-cancer agent in apoptosis-resistant cancer cells. 

## 4. Materials and Methods

### 4.1. Chemicals and Reagents

The DK from *E. cava* was prepared in previous our studies [20,23,25]. The MTT was purchased from Sigma Chemical Co. (St. Louis, MO, USA). The Z-VAD-fmk, 3MA, HCQ, E64D, and pepstatin A were purchased from Selleck Chemicals (Houston, TX, USA). The primary antibodies against caspase-3 (#2696), cleaved caspase-3 (#9664), PARP (#9542), cleaved PARP (#5625), Bcl-2 (#4223), Bax (#5023), Bim (#2933), Bak (#12105), LC3B (#3868) were purchased from Cell Signaling Technology (Danvers, MA, USA). Sequestosome 1 (SQSTM1, p62, sc-28359) and LAMP-1, (sc-20011) were purchased from Santa Cruz (St. Dallas, TX, USA), and actin (MAB1501) was purchased from Millipore (Billerica, MA, USA). All the remaining chemicals and reagents were purchased from Sigma-Aldrich (Merck Millipore, Darmstadt, Germany).

### 4.2. Cell Culture and MTT Assay

The A375 human melanoma cells were obtained from American Type Culture Collection (ATCC, Manassas, VA, USA) and cultured in Dulbecco’s modified Eagle medium (DMEM, Welgene, Republic of Korea) containing 10% fetal bovine serum (FBS, Welgene, Republic of Korea), 100 U/mL of penicillin, and 100 μg/mL of streptomycin in an incubator with 5% CO_2_ at 37 °C. The anti-growth effect of DK was measured using the MTT assay. The A375 cells were seeded at a density of 4 × 10^3^ cells/well in 96-well plates. After 24 h of seeding, the cells were incubated with vehicle dimethyl sulfoxide (DMSO) or various concentrations of DK for 48 h. Thereafter, the cells were treated with the MTT solution (1 mg/mL final concentration) and incubated for 4 h at 37 °C. The resulting formazan crystals were dissolved in 100 μL DMSO. Absorbance was determined at 595 nm using a microplate reader (FilterMax F5, Molecular Devices) (Molecular Devices, LLC, Sunnyvale, CA, USA).

### 4.3. Colony Formation Assay

For the colony formation assay, A375 cells were seeded in 24-well plates at 5 × 10^3^ cells/well. After incubation for 24 h, cells were treated with vehicle DMSO, DK, or other compounds for 48 h and then incubated with DMEM for an additional 5 days. Finally, the cells were washed with phosphate-buffered saline (PBS), stained with 0.5% crystal violet for 10 min, and photographed under a microscope (Motic AE31, MHG-100B; Jed Pella Co., Redding, CA, USA; DM3000; Leica, Wetzlar, Germany).

### 4.4. Apoptosis Assay

Cells were seeded at a density of 4.6 × 10^4^ cells/well in a 12-well plate. After incubation for 24 h, the cells were treated with vehicle DMSO or DK for 48 h. The cells were washed twice with PBS, adjusted to 100 μL of solution and stained with Annexin V-FITC and PI using the FITC Annexin V Apoptosis Detection Kit (BD Biosciences, San Jose, CA, USA). The cells were incubated for 10 min at room temperature (RT). The cells were analyzed using a FACSVerse^TM^ flow cytometer (BD Bioscience, Franklin Lakes, NJ, USA) (Ex/Em = 488 nm/530 nm) and data was calculated using flowJo^TM^ Software (BD Bioscience, Franklin Lakes, NJ, USA).

### 4.5. Immunofluorescence

Cells were seeded on coverslips and treated with DMSO, DK, or HCQ washed once with ice-cold PBS and then fixed using 4% paraformaldehyde for 5 min at room temperature. After fixation, the cells were washed thrice with PBS and then permeabilized with 0.1% *v/v* Triton X-100 for 5 min and washed three times with PBS. A PBS blocking solution containing 1% bovine serum albumin (BSA) was then added onto the coverslips. After 30 min of incubation, appropriately diluted primary and secondary antibodies were incubated for 1 h at RT. The coverslips were washed thrice with PBS and mounted on glass slides using Pro-Long gold anti-fade mounting medium (Invitrogen, Waltham, MA, USA). The coverslips were then imaged using a Zeiss LSM780 confocal microscope (Zeiss LSM 780, Oberkochen, Germany) with a 40× oil-free immersion objective.

### 4.6. LMP Assay

Lysosomal membrane permeabilization was assessed by acridine orange (AO) staining. The AO exhibits red fluorescence at high concentrations, similar to lysosomes, whereas green fluorescence occurs in the cytosol at low concentrations. The cells were seeded onto coverslips in 24-well plates. After incubation for 24 h, the cells were treated with vehicle DMSO, DK, or HCQ for 24 h. The cells were then stained with 5 mg/mL AO (Thermo Fisher Scientific, Waltham, MA, USA) at 37 °C for 30 min, rinsed with ice-cold PBS, and observed under a fluorescence microscope (Zeiss LSM 780, Oberkochen, Germany).

### 4.7. Mitochondrial Membrane Potential Assay

Mitochondrial membrane potential was determined using the mitochondrial membrane potential detection JC-1 kit (BD Bioscience) according to the manufacturer’s protocol. Cells (4.6 × 10^4^ cells/well) were seeded in 12-well plates and treated with compound for 48 h. The collected cells were incubated with JC-1 at 37 °C for 20 min and washed twice with 1 × assay buffer. Changes in the mitochondrial membrane potential were detected using a FACSVerse^TM^ flow cytometer (Ex/Em = 514 nm/Em 529~590 nm) (BD Biosciences, Franklin, Lakes, NJ, USA).

### 4.8. Cathepsin Activity Assay

Cathepsin activity was determined using a fluorescence-based CTSB and CTSD activity assay kit (Sigma; Merck Millipore, Darmstadt, Germany). according to the manufacturer’s protocol. Briefly, cells were incubated with vehicle DMSO or DK for 48 h, and cell lysates were prepared. Cathepsin activity was measured using a 0.02 mM CTSB substrate (Z-Arg-Arg-AMC) or CTSD substrates (GKPILFFRLK(Dnp)-DR-NH2). The cleavage of the synthetic substrate was quantified at an excitation wavelength of 320 nm and emission wavelength of 535 nm using a microplate reader. Cathepsin activity was expressed as relative fluorescence units (RFU) per microgram of protein.

### 4.9. Western Blot Analysis

Western blotting was performed according to standard procedures. Briefly, A375 cells (4.6 × 10^4^ cells/well) were seeded in 12-well plates with serum-free media. After incubation for 24 h, the cells were treated with vehicle DMSO, DK, or other compounds for 48 h. Cells were lysed in radioimmunoprecipitation assay (RIPA) buffer at 4 °C. Total proteins were extracted, and 100 μg/mL of protein was separated using 8–10% sodium dodecyl sulfate (SDS)-polyacrylamide gels and transferred to polyvinylidene difluoride (PVDF) membranes (Amersham Pharmacia Biotech, England, UK). The reaction was blocked with 5% skim milk and 1% BSA in PBS for 1.5 h at RT, and probed with primary antibodies and secondary antibodies. The respective proteins were detected using a chemiluminescent ECL assay kit (Amersham Pharmacia, UK). The immunoblotted bands were visualized using an LAS-3000 system and quantified using MultiGauge V 3.0 software (Fujifilm Life Science, Tokyo, Japan).

### 4.10. Statistical Analysis

All data were analyzed using Instat statistics program (GraphPad Software, Inc., San Diego, CA, USA). Statistical comparisons were performed using one-way analysis of variance (ANOVA) with the Bonferroni multiple comparison test. All results are shown as the mean ± standard deviation (SD) of at least three independent experiments. *** *p* < 0.001, ** *p* < 0.01, * *p* < 0.05 compared to the control.

## 5. Conclusions

These results suggest that dieckol induces apoptotic cell death and inhibits the growth of A375 human melanoma cells via inhibition of autophagic flux, induction of lysosomal dysfunction, and loss of mitochondria membrane potential. Therefore, dieckol may be a novel chemotherapeutic autophagy inhibitor used against human melanoma. A more detailed study of the mechanism by which dieckol induces LMP and MMP is required for developing therapeutic strategies for the treatment of melanoma.

## Data Availability

The data that support the findings of this study are available from the corresponding author (S.J.P.) upon reasonable request.
